# Low-grade appendiceal neoplasm presenting as a volvulus of the cecum

**DOI:** 10.1093/gastro/got026

**Published:** 2013-10-08

**Authors:** Vincent Costa, Jonas P. DeMuro

**Affiliations:** ^1^School of Medicine, Stony Brook University, Stony Brook, NY, USA and ^2^Department of Surgery, Winthrop University Hospital, Mineola, NY, USA

**Keywords:** surgical resection, appendix, appendix carcinoma

## Abstract

Mucocele of the appendix is an uncommon disorder that is often asymptomatic, but can present similarly to acute appendicitis. Timely diagnosis and treatment is imperative due to the many complications that can result from the mucocele, such as perforation. Appendiceal mucoceles (AM) were previously thought to be either benign or malignant; however, a different pathological classification of AM is currently favored. Also, only a few cases of volvulus of a benign AM have been reported. Here, we present the first reported case of a low-grade appendiceal mucinous neoplasm resulting in a volvulus of the cecum.

## INTRODUCTION

Mucoceles of the appendix are a rare entity, as they represent only 0.2–0.3% of all surgical appendectomy specimens [1]. Although mucoceles of the appendix are infrequent, appendicitis is so common in the general population that it is probable that a surgeon will encounter a mucocele only a few times in his or her career. Appendiceal mucoceles (AM) can present in a variety of ways, ranging from acute appendicitis-like symptoms to a palpable mass in the right lower quadrant. In fact, up to 50% of mucoceles are found incidentally during radiological evaluations, endoscopic procedures or surgery [2]. Of those patients proven to have a mucocele during surgery, approximately 40% were diagnosed pre-operatively as having acute appendicitis on clinical grounds alone. Recently, pathologists have changed the way they are classifying these neoplasms. At one time, mucoceles of the appendix were considered either benign or malignant. However, today, pathologists are favoring other terms that take into account the wide range of pathological severity. Their clinical significance lies with the variety of complications that can ensue. The most common and dangerous complication is rupture, with subsequent spillage of tumor into the peritoneal cavity, resulting in pseudomyxoma peritonei [1, 2].

The most common portion of the colon to have a volvulus is in the sigmoid portion, with cecal volvulus occurring less commonly. Cecal volvulus can be divided into three types: axial torsion (Type I), loop type (Type II) and cecal bascule (Type III). It occurs when there is a lack of peritoneal fixation of the cecum, allowing it to be more mobile and resulting in the obstruction. It is extremely rare for a mucocele to present with the complication of a cecal volvulus. Here we present the first case of a low-grade appendiceal mucinous neoplasm causing a volvulus of the cecum.

## CASE REPORT

A 77-year-old male presented to our Emergency Department with a one-day history of abdominal pain located in the right lower quadrant. The pain was sharp and of sudden onset; however he denied any fevers, nausea, vomiting or changes in bowel habits. His medical history was significant for hypertension and chronic renal insufficiency. The surgical history included an aortic valve replacement and an open repair of an abdominal aortic aneurysm 10 years previously.

On examination, he had a temperature of 99.0°F and a blood pressure of 157/86; the remaining vital signs were normal. The abdominal exam was significant for tenderness in the right lower quadrant, with a palpable mass. There was a small incisional hernia superior to the umbilicus from his prior abdominal aortic aneurysm repair, which was non-incarcerated. There were no peritoneal signs, and the rest of the abdomen was benign.

**Fig. 1.**
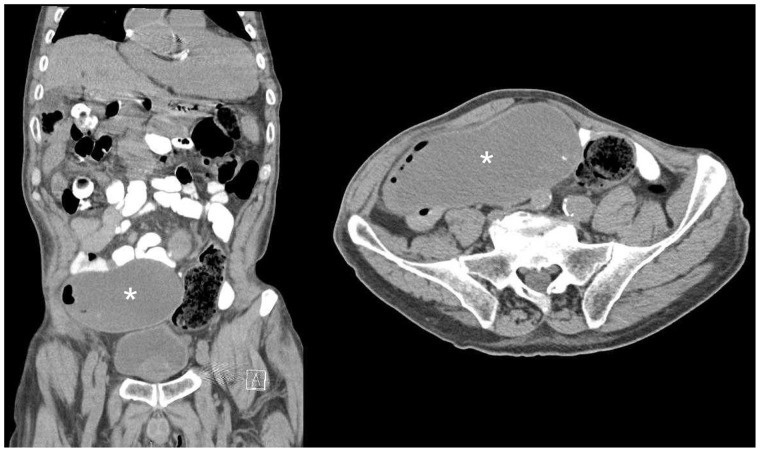
Computed tomography of the abdomen and pelvis, with oral contrast only in both the coronal and axial images. A white asterisk (*) in both views indicates the low-grade mucinous neoplasm of the appendix.

Laboratory work-up revealed a white blood cell count of 8000/µL with 79% neutrophils. The blood urea nitrogen and creatinine were at his baseline of 33 mg/dL and 1.7 mg/dL respectively, consistent with his chronic renal failure. In addition, his carcinoembryonic antigen (CEA) level was mildly elevated at 4 ng/mL. The remainder of the laboratory work-up, including electrolytes, hemoglobin and hepatic profile, were normal. A computed tomography (CT) scan without contrast of the abdomen and pelvis was obtained, which showed a large, thin-walled structure containing hypodense material in the right lower quadrant (Fig. 1). The structure was contiguous with the base of the cecum, which made the findings most consistent with an AM. There was also rotation of the ascending colon along its axis, which caused luminal narrowing, with the mucocele acting as the fulcrum for a volvulus of the cecum (Type I).

**Fig. 2.**
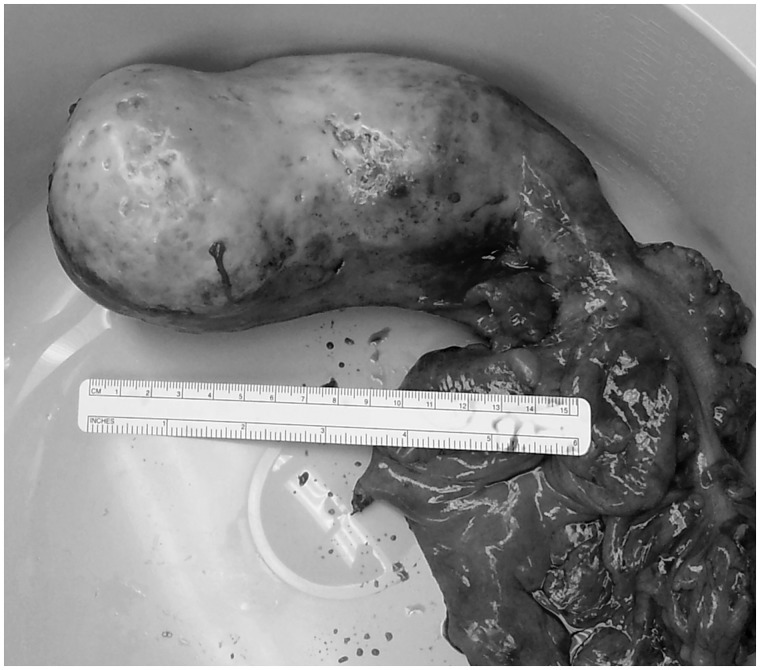
Specimen photo shows the mass of the appendix, and the right colon with mesentery.

The patient was brought to the operating room as an emergency case and a midline incision was made for an exploratory laparotomy. A large appendiceal mass was identified, which extended to the cecal base (Fig. 2). The AM had rotated, such that when the mucocele twisted, it caused a volvulus of the cecum. Due to the large size of this mass and involvement of the cecum, a formal right hemicolectomy was initiated. The right colon was mobilized and resected from the terminal ileum to the transverse colon, taking care to be atraumatic, with no rupture of the specimen. A primary anastomosis was performed with a stapled side-to-side technique.

The pathology report described a cystically dilated and enlarged appendix measuring 17 cm in length and ranging in diameter from 2.8 cm at the appendiceal orifice to a maximal diameter of 7.5 cm at the tip. The epithelium of the appendix was mucinous, flat, and showed low-grade dysplasia. The histological features are consistent with low-grade appendiceal mucinous neoplasm. Focally acellular mucin dissection into the extensively fibrotic wall of the appendix was seen. In addition, the tumor extended from the appendix and was found in the cecum, at the junction of the appendix and cecum. The resection margins were negative for tumor or dysplasia and 36 lymph nodes were negative for tumor. The patient’s post-operative course was complicated by a seizure on post-op day two. It was treated with medical management and the remainder of the post-operative course was uneventful. The patient was discharged on post-operative day nine, and made a full recovery on outpatient follow-up.

## DISCUSSION

Mucocele of the appendix was first described by Rokitansky in 1842 and then definitively named by Feren in 1876 [2]. Since then, there has been debate as to how these mucoceles arise. There are two possible mechanisms for mucocele formation: (i) an obstructive process that causes elevated appendiceal pressures, leading to gross mucinous accumulation or (ii) strictly a neoplastic process [3]. The latter description encompasses most AM diagnoses and applies to how an appendiceal neoplastic process may spread to the lymph nodes, or extend into the surrounding tissues or even seed into the peritoneum. Regarding the size of AMs, those greater than 6 cm have a reported rupture rate above 20% [4]. This emphasizes the importance of timely diagnosis and proper surgical removal.

Mucocele of the appendix is a rare entity that can present with a variety of symptoms, or be an incidental surgical finding. It is a progressive dilation of the appendix due to mucus accumulation and makes up only 0.2–0.3% of all appendiceal specimens [1]. They have a higher frequency of occurrence in women, with a ratio of 4:1 between women and men, although this predominance has been challenged in more recent studies [5]. In addition, over 75% of cases occur in persons older than 50 years [6]. Over 50% of all cases of mucocele were clinically asymptomatic, which emphasizes the important role that radiologic findings play in the diagnosis of AMs. Fine needle aspiration of the mucocele is contra-indicated, due to the risk of dissemination of the mucus into the peritoneum. Both ultrasound and CT scan findings in mucoceles of the appendix have been clearly defined [7]. Ultrasound examination can show a cystic mass with posterior enhancement, poorly defined walls and variable internal echogenicity. Giant mucoceles of the appendix can even show the typical, pathognomonic ‘onion skin sign’. CT of the abdomen and pelvis is the best study method for the diagnosis of AM. It can show a well encapsulated structure with low attenuation in the appendix [8]. In our patient, the CT scan helped us determine whether the palpable mass in the right lower quadrant was caused by an inflamed appendix or an appendiceal neoplasm.

In addition, it is important to keep in mind that appendiceal mucinous neoplasms are associated with an increased incidence of other tumors. In some reports, the rate of synchronous tumors is as high as 29% [9]. The most frequently associated neoplasms are colon and rectal cancers, followed by epithelial ovarian cancer. AMs are nearly always present in women who are diagnosed with ovarian cystadenocarcinoma [10]. Follow-up colonoscopy and pelvic exams of these patients have been shown to have practical value in their management post-operatively. In addition, it is important to obtain a baseline CEA level and monitor its progression post-operatively. Our patient had a mildly elevated CEA level of 4.0 ng/mL at the time of surgery. It is important to follow his CEA levels, for an increase in CEA may signify recurrence or a commonly occurring synchronous tumor development [11].

An AM can be clinically confused with acute appendicitis, which is why pre-operative diagnosis is important to determine the surgical approach. The most common presentation of symptomatic mucocele of the appendix is acute or chronic right lower quadrant pain. Nausea and vomiting, as well as a change in bowel habits, have also been reported. An intra-abdominal mass can be palpated by the physician on examination in about half of these cases [12]. Open surgical techniques have traditionally been performed for these tumors to prevent dissemination of the mucocele into the peritoneal cavity. Of primary concern to the surgeon is the prevention of dissemination of the mucocele into the peritoneal cavity. Laparoscopic dissection, by grasping of the mucocele and pneumoperitoneum, and transporting the specimen through the abdominal wall, may contribute to peritoneal dissemination of an appendiceal mucinous tumor [13]. However, right hemicolectomy, while the ‘gold standard’ operation for a malignancy of the appendix, is not always required in the management of mucinous appendiceal malignancies, with select tumors amenable to minimally invasive surgery. For tumors that are proximal to the appendix, or involve the cecum, a right hemicolectomy is advocated, while distal smaller tumors may be treated with only an appendectomy, pending pathological results of clear margins [14].

The classification of appendiceal mucinous neoplasms is controversial, and has recently been altered. The former classification included histological categories including mucinous cystadenoma and mucinous cystadenocarcinoma, commonly distinguished by the presence of tumor extension into the peritoneum [15]. In 1995, Carr and co-authors introduced a new classification for AMs called mucinous tumors of uncertain malignant potential (UMP) [16]. These were dysplastic tumors that are difficult to classify as clearly benign or malignant. However, a mucinous tumor of UMP was not universally accepted, because it created gray zones that were difficult to resolve. For example, many mucinous tumors of UMP were located in the appendix, some had cells spread to the right lower quadrant, and some had cells that invaded the ovaries—yet they were all classified as UMP. Many pathologists argue that appendiceal tumors should be classified based on their histology, such as degree of dysplasia, rather than location of spread. This is because many appendiceal tumors that are confined to the appendix are indistinguishable from those that have spread to the peritoneum. Pathologists are currently favoring the intermediate term ‘low-grade appendiceal mucinous neoplasm’, a term used to encompass low-grade appendiceal mucinous tumors regardless of their stage [17]. This approach conforms to the manner by which other tumors are classified. Due to this new classification, the 5-year and 10-year survivals of patients with low-grade appendiceal mucinous neoplasms is not known and we advocate the long-term follow-up of these patients to quantify their outcomes.

Appendiceal mucocele is a difficult diagnosis to make clinically. However, its importance lies in the potential complications that can follow. The most common complication is perforation, which has an incidence of up to 20%. Rupture with subsequent pseudomyxoma peritonei is the most feared outcome in this scenario [18]; this occurs when mucinous epithelial cells from a ruptured AM implant onto the peritoneal surface. These neoplastic mucinous cells can fill the abdominal cavity and cause fibrosis of surrounding tissues and organs [19]. If left untreated, tumors and mucin will eventually build up and begin to compress essential structures such as the colon, small intestine or kidneys. They can also form adhesions that may cause intestinal obstruction and carcinomatosis [20]. Similarly to the classification of AMs, there is substantial debate regarding the histopathological classification of pseudomyxoma peritonei [21].

Other complications of AMs have also been described. Albeit much less common, these complications include intestinal obstructions, intussusception, intestinal bleeding, fistula formation and volvulus. It is extremely uncommon for an AM to cause a volvulus, with few cases reported since its initial description in 1946 [9]. However, on literature search there are no previously published reports of a low-grade appendiceal mucinous neoplasm acting as the lead point for a volvulus of the cecum, making this case unique.

**Conflict of interest:** none declared.
